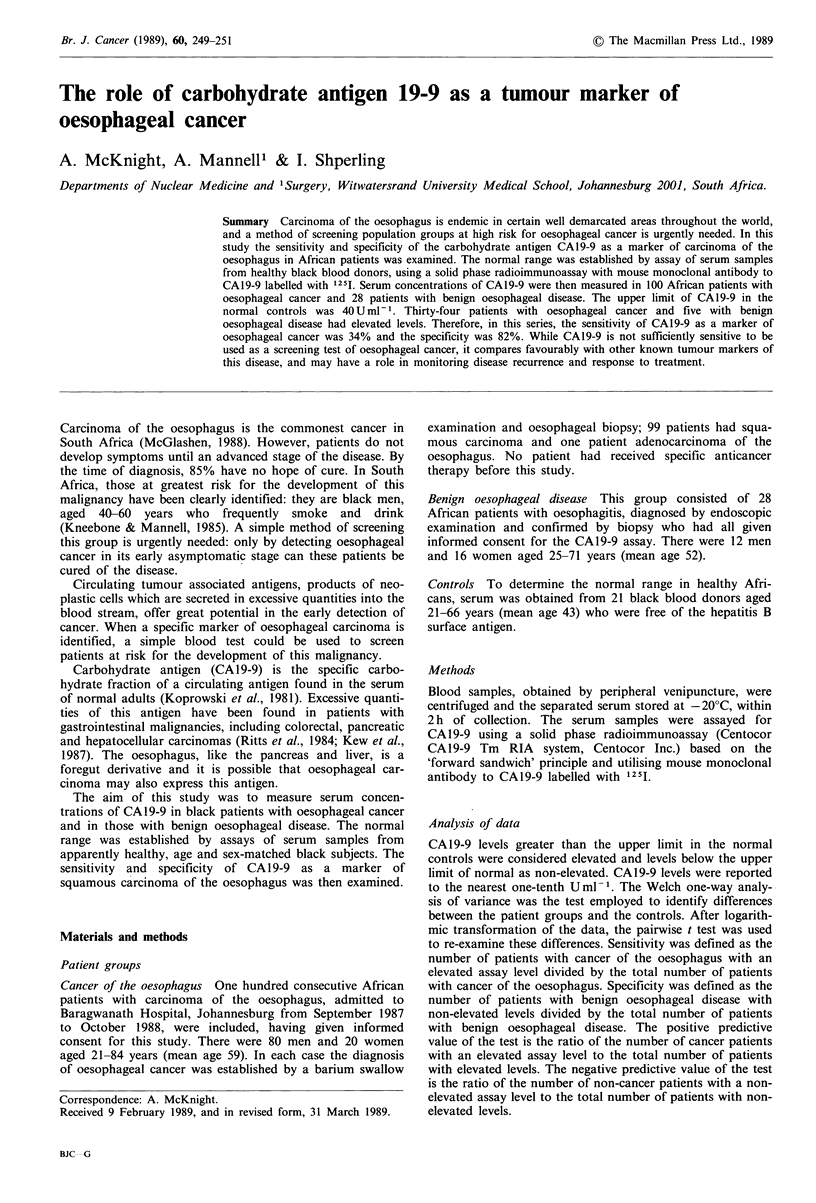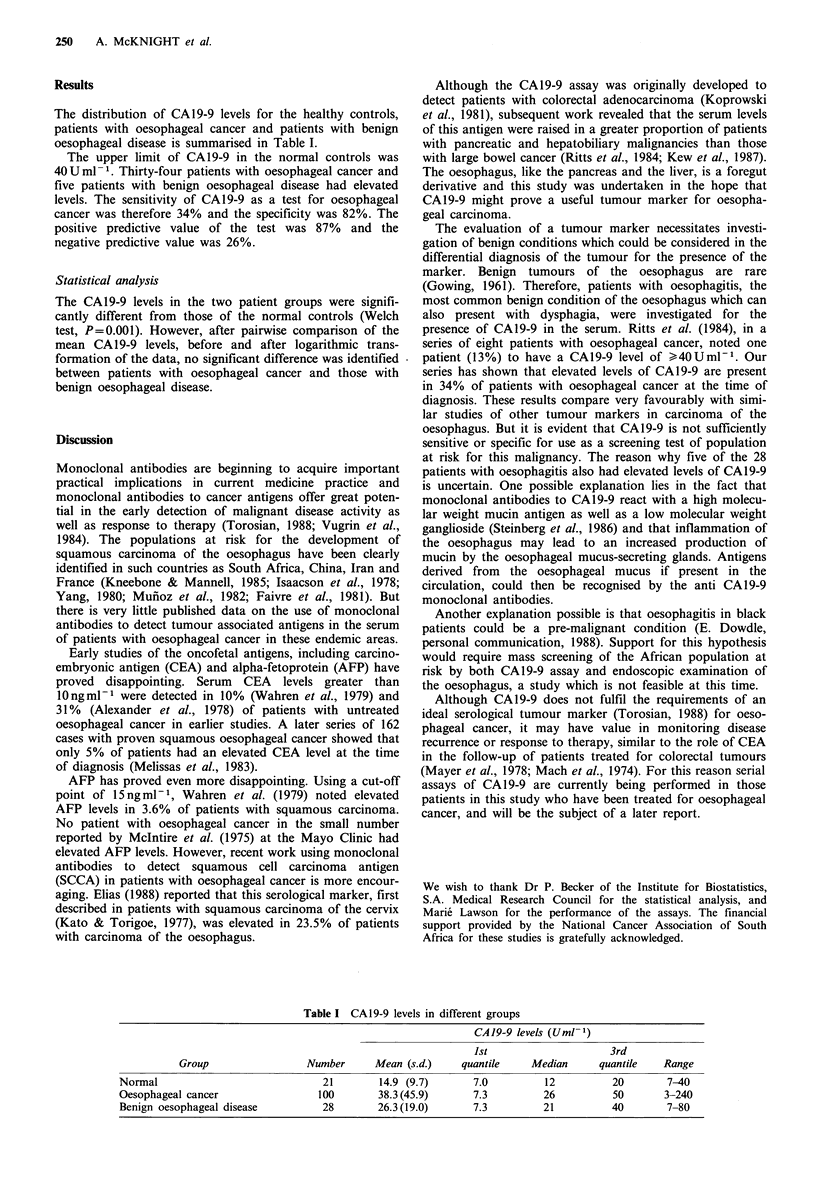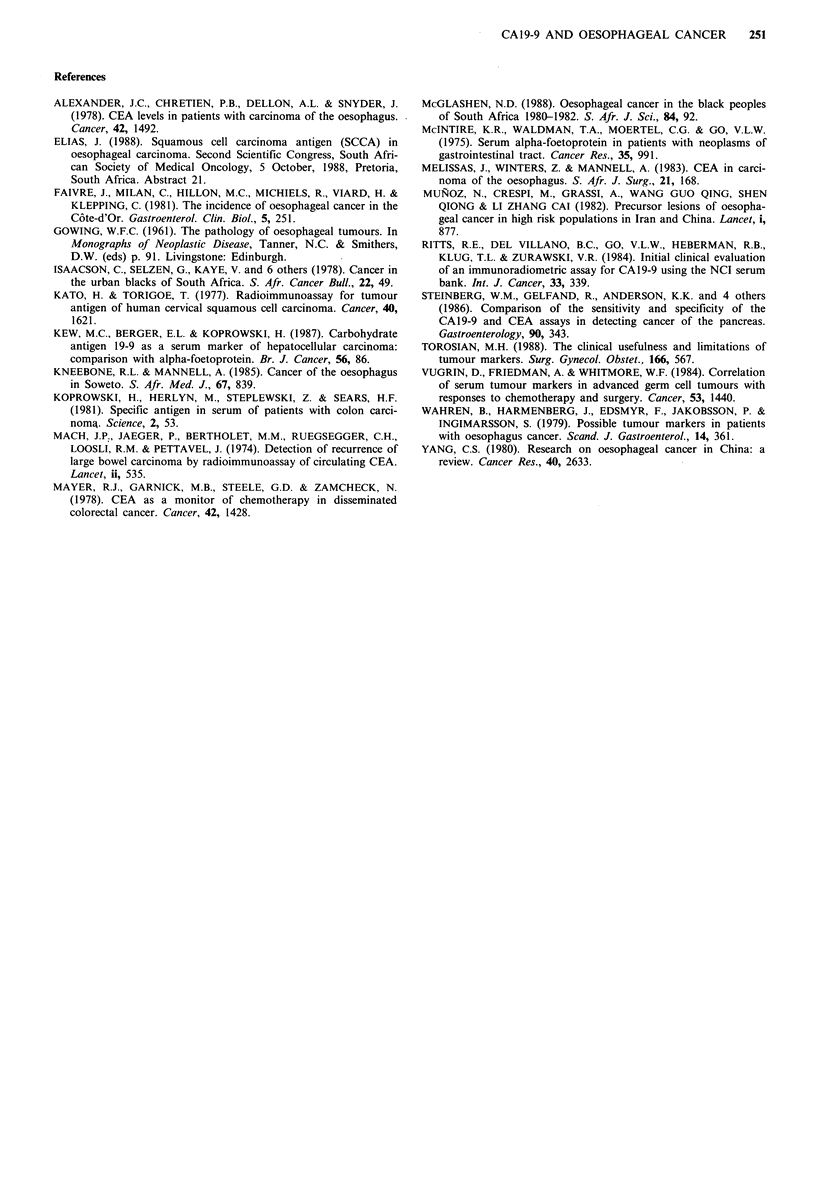# The role of carbohydrate antigen 19-9 as a tumour marker of oesophageal cancer.

**DOI:** 10.1038/bjc.1989.263

**Published:** 1989-08

**Authors:** A. McKnight, A. Mannell, I. Shperling

**Affiliations:** Department of Nuclear Medicine, Witwatersrand University Medical School, Johannesburg, South Africa.

## Abstract

Carcinoma of the oesophagus is endemic in certain well demarcated areas throughout the world, and a method of screening population groups at high risk for oesophageal cancer is urgently needed. In this study the sensitivity and specificity of the carbohydrate antigen CA19-9 as a marker of carcinoma of the oesophagus in African patients was examined. The normal range was established by assay of serum samples from healthy black blood donors, using a solid phase radioimmunoassay with mouse monoclonal antibody to CA19-9 labelled with 125I. Serum concentrations of CA19-9 were then measured in 100 African patients with oesophageal cancer and 28 patients with benign oesophageal disease. The upper limit of CA19-9 in the normal controls was 40 U ml-1. Thirty-four patients with oesophageal cancer and five with benign oesophageal disease had elevated levels. Therefore, in this series, the sensitivity of CA19-9 as a marker of oesophageal cancer was 34% and the specificity was 82%. While CA19-9 is not sufficiently sensitive to be used as a screening test of oesophageal cancer, it compares favourably with other known tumour markers of this disease, and may have a role in monitoring disease recurrence and response to treatment.


Br. J. Cancer (1989), 60, 249-251 © The Macmillan Press Ltd., 1989

### The role of carbohydrate antigen 19-9 as a tumour marker of oesophageal cancer

A. McKnight, A. Mannell[1] & I. Shperling

*Departments of Nuclear Medicine and [1]Surgery, Witwatersrand University Medical School, Johannesburg 2001, South Africa.*

**Summary** Carcinoma of the oesophagus is endemic in certain well demarcated areas throughout the world, and a method of screening population groups at high risk for oesophageal cancer is urgently needed. In this study the sensitivity and specificity of the carbohydrate antigen CA19-9 as a marker of carcinoma of the oesophagus in African patients was examined. The normal range was established by assay of serum samples from healthy black blood donors, using a solid phase radioimmunoassay with mouse monoclonal antibody to CA19-9 labelled with $^{125}I$. Serum concentrations of CA19-9 were then measured in 100 African patients with oesophageal cancer and 28 patients with benign oesophageal disease. The upper limit of CA19-9 in the normal controls was $40\,U\,ml^{-1}$. Thirty-four patients with oesophageal cancer and five with benign oesophageal disease had elevated levels. Therefore, in this series, the sensitivity of CA19-9 as a marker of oesophageal cancer was 34% and the specificity was 82%. While CA19-9 is not sufficiently sensitive to be used as a screening test of oesophageal cancer, it compares favourably with other known tumour markers of this disease, and may have a role in monitoring disease recurrence and response to treatment.

Carcinoma of the oesophagus is the commonest cancer in South Africa (McGlashen, 1988). However, patients do not develop symptoms until an advanced stage of the disease. By the time of diagnosis, 85% have no hope of cure. In South Africa, those at greatest risk for the development of this malignancy have been clearly identified: they are black men, aged 40–60 years who frequently smoke and drink (Kneebone & Mannell, 1985). A simple method of screening this group is urgently needed: only by detecting oesophageal cancer in its early asymptomatic stage can these patients be cured of the disease.

Circulating tumour associated antigens, products of neoplastic cells which are secreted in excessive quantities into the blood stream, offer great potential in the early detection of cancer. When a specific marker of oesophageal carcinoma is identified, a simple blood test could be used to screen patients at risk for the development of this malignancy.

Carbohydrate antigen (CA19-9) is the specific carbohydrate fraction of a circulating antigen found in the serum of normal adults (Koprowski *et al.*, 1981). Excessive quantities of this antigen have been found in patients with gastrointestinal malignancies, including colorectal, pancreatic and hepatocellular carcinomas (Ritts *et al.*, 1984; Kew *et al.*, 1987). The oesophagus, like the pancreas and liver, is a foregut derivative and it is possible that oesophageal carcinoma may also express this antigen.

The aim of this study was to measure serum concentrations of CA19-9 in black patients with oesophageal cancer and in those with benign oesophageal disease. The normal range was established by assays of serum samples from apparently healthy, age and sex-matched black subjects. The sensitivity and specificity of CA19-9 as a marker of squamous carcinoma of the oesophagus was then examined.

### Materials and methods

### *Patient groups*

*Cancer of the oesophagus* One hundred consecutive African patients with carcinoma of the oesophagus, admitted to Baragwanath Hospital, Johannesburg from September 1987 to October 1988, were included, having given informed consent for this study. There were 80 men and 20 women aged 21–84 years (mean age 59). In each case the diagnosis of oesophageal cancer was established by a barium swallow examination and oesophageal biopsy; 99 patients had squamous carcinoma and one patient adenocarcinoma of the oesophagus. No patient had received specific anticancer therapy before this study.

*Benign oesophageal disease* This group consisted of 28 African patients with oesophagitis, diagnosed by endoscopic examination and confirmed by biopsy who had all given informed consent for the CA19-9 assay. There were 12 men and 16 women aged 25–71 years (mean age 52).

*Controls* To determine the normal range in healthy Africans, serum was obtained from 21 black blood donors aged 21–66 years (mean age 43) who were free of the hepatitis B surface antigen.

### *Methods*

Blood samples, obtained by peripheral venipuncture, were centrifuged and the separated serum stored at −20°C, within 2 h of collection. The serum samples were assayed for CA19-9 using a solid phase radioimmunoassay (Centocor CA19-9 Tm RIA system, Centocor Inc.) based on the 'forward sandwich' principle and utilising mouse monoclonal antibody to CA19-9 labelled with $^{125}I$.

### *Analysis of data*

CA19-9 levels greater than the upper limit in the normal controls were considered elevated and levels below the upper limit of normal as non-elevated. CA19-9 levels were reported to the nearest one-tenth $U\,ml^{-1}$. The Welch one-way analysis of variance was the test employed to identify differences between the patient groups and the controls. After logarithmic transformation of the data, the pairwise $t$ test was used to re-examine these differences. Sensitivity was defined as the number of patients with cancer of the oesophagus with an elevated assay level divided by the total number of patients with cancer of the oesophagus. Specificity was defined as the number of patients with benign oesophageal disease with non-elevated levels divided by the total number of patients with benign oesophageal disease. The positive predictive value of the test is the ratio of the number of cancer patients with an elevated assay level to the total number of patients with elevated levels. The negative predictive value of the test is the ratio of the number of non-cancer patients with a non-elevated assay level to the total number of patients with non-elevated levels.

Correspondence: A. McKnight.
Received 9 February 1989, and in revised form, 31 March 1989.

### Results

The distribution of CA19-9 levels for the healthy controls, patients with oesophageal cancer and patients with benign oesophageal disease is summarised in Table I.

The upper limit of CA19-9 in the normal controls was $40\,U\,ml^{-1}$. Thirty-four patients with oesophageal cancer and five patients with benign oesophageal disease had elevated levels. The sensitivity of CA19-9 as a test for oesophageal cancer was therefore 34% and the specificity was 82%. The positive predictive value of the test was 87% and the negative predictive value was 26%.

### *Statistical analysis*

The CA19-9 levels in the two patient groups were significantly different from those of the normal controls (Welch test, $P=0.001$). However, after pairwise comparison of the mean CA19-9 levels, before and after logarithmic transformation of the data, no significant difference was identified between patients with oesophageal cancer and those with benign oesophageal disease.

### Discussion

Monoclonal antibodies are beginning to acquire important practical implications in current medicine practice and monoclonal antibodies to cancer antigens offer great potential in the early detection of malignant disease activity as well as response to therapy (Torosian, 1988; Vugrin *et al.*, 1984). The populations at risk for the development of squamous carcinoma of the oesophagus have been clearly identified in such countries as South Africa, China, Iran and France (Kneebone & Mannell, 1985; Isaacson *et al.*, 1978; Yang, 1980; Muñoz *et al.*, 1982; Faivre *et al.*, 1981). But there is very little published data on the use of monoclonal antibodies to detect tumour associated antigens in the serum of patients with oesophageal cancer in these endemic areas.

Early studies of the oncofetal antigens, including carcinoembryonic antigen (CEA) and alpha-fetoprotein (AFP) have proved disappointing. Serum CEA levels greater than $10\,ng\,ml^{-1}$ were detected in 10% (Wahren *et al.*, 1979) and 31% (Alexander *et al.*, 1978) of patients with untreated oesophageal cancer in earlier studies. A later series of 162 cases with proven squamous oesophageal cancer showed that only 5% of patients had an elevated CEA level at the time of diagnosis (Melissas *et al.*, 1983).

AFP has proved even more disappointing. Using a cut-off point of $15\,ng\,ml^{-1}$, Wahren *et al.* (1979) noted elevated AFP levels in 3.6% of patients with squamous carcinoma. No patient with oesophageal cancer in the small number reported by McIntire *et al.* (1975) at the Mayo Clinic had elevated AFP levels. However, recent work using monoclonal antibodies to detect squamous cell carcinoma antigen (SCCA) in patients with oesophageal cancer is more encouraging. Elias (1988) reported that this serological marker, first described in patients with squamous carcinoma of the cervix (Kato & Torigoe, 1977), was elevated in 23.5% of patients with carcinoma of the oesophagus.

Although the CA19-9 assay was originally developed to detect patients with colorectal adenocarcinoma (Koprowski *et al.*, 1981), subsequent work revealed that the serum levels of this antigen were raised in a greater proportion of patients with pancreatic and hepatobiliary malignancies than those with large bowel cancer (Ritts *et al.*, 1984; Kew *et al.*, 1987). The oesophagus, like the pancreas and the liver, is a foregut derivative and this study was undertaken in the hope that CA19-9 might prove a useful tumour marker for oesophageal carcinoma.

The evaluation of a tumour marker necessitates investigation of benign conditions which could be considered in the differential diagnosis of the tumour for the presence of the marker. Benign tumours of the oesophagus are rare (Gowing, 1961). Therefore, patients with oesophagitis, the most common benign condition of the oesophagus which can also present with dysphagia, were investigated for the presence of CA19-9 in the serum. Ritts *et al.* (1984), in a series of eight patients with oesophageal cancer, noted one patient (13%) to have a CA19-9 level of $\geqslant 40\,U\,ml^{-1}$. Our series has shown that elevated levels of CA19-9 are present in 34% of patients with oesophageal cancer at the time of diagnosis. These results compare very favourably with similar studies of other tumour markers in carcinoma of the oesophagus. But it is evident that CA19-9 is not sufficiently sensitive or specific for use as a screening test of population at risk for this malignancy. The reason why five of the 28 patients with oesophagitis also had elevated levels of CA19-9 is uncertain. One possible explanation lies in the fact that monoclonal antibodies to CA19-9 react with a high molecular weight mucin antigen as well as a low molecular weight ganglioside (Steinberg *et al.*, 1986) and that inflammation of the oesophagus may lead to an increased production of mucin by the oesophageal mucus-secreting glands. Antigens derived from the oesophageal mucus if present in the circulation, could then be recognised by the anti CA19-9 monoclonal antibodies.

Another explanation possible is that oesophagitis in black patients could be a pre-malignant condition (E. Dowdle, personal communication, 1988). Support for this hypothesis would require mass screening of the African population at risk by both CA19-9 assay and endoscopic examination of the oesophagus, a study which is not feasible at this time.

Although CA19-9 does not fulfil the requirements of an ideal serological tumour marker (Torosian, 1988) for oesophageal cancer, it may have value in monitoring disease recurrence or response to therapy, similar to the role of CEA in the follow-up of patients treated for colorectal tumours (Mayer *et al.*, 1978; Mach *et al.*, 1974). For this reason serial assays of CA19-9 are currently being performed in those patients in this study who have been treated for oesophageal cancer, and will be the subject of a later report.

We wish to thank Dr P. Becker of the Institute for Biostatistics, S.A. Medical Research Council for the statistical analysis, and Marié Lawson for the performance of the assays. The financial support provided by the National Cancer Association of South Africa for these studies is gratefully acknowledged.

**Table I** CA19-9 levels in different groups

| Group | Number | *CA19-9 levels* ($U\,ml^{-1}$) Mean (s.d.) | 1st quantile | Median | 3rd quantile | Range |
|---|---|---|---|---|---|---|
| Normal | 21 | 14.9 (9.7) | 7.0 | 12 | 20 | 7–40 |
| Oesophageal cancer | 100 | 38.3 (45.9) | 7.3 | 26 | 50 | 3–240 |
| Benign oesophageal disease | 28 | 26.3 (19.0) | 7.3 | 21 | 40 | 7–80 |

### References

ALEXANDER, J.C., CHRETIEN, P.B., DELLON, A.L. & SNYDER, J. (1978). CEA levels in patients with carcinoma of the oesophagus. *Cancer*, **42**, 1492.

ELIAS, J. (1988). Squamous cell carcinoma antigen (SCCA) in oesophageal carcinoma. Second Scientific Congress, South African Society of Medical Oncology, 5 October, 1988, Pretoria, South Africa. Abstract 21.

FAIVRE, J., MILAN, C., HILLON, M.C., MICHIELS, R., VIARD, H. & KLEPPING, C. (1981). The incidence of oesophageal cancer in the Côte-d'Or. *Gastroenterol. Clin. Biol.*, **5**, 251.

GOWING, W.F.C. (1961). The pathology of oesophageal tumours. In *Monographs of Neoplastic Disease*, Tanner, N.C. & Smithers, D.W. (eds) p. 91. Livingstone: Edinburgh.

ISAACSON, C., SELZEN, G., KAYE, V. and 6 others (1978). Cancer in the urban blacks of South Africa. *S. Afr. Cancer Bull.*, **22**, 49.

KATO, H. & TORIGOE, T. (1977). Radioimmunoassay for tumour antigen of human cervical squamous cell carcinoma. *Cancer*, **40**, 1621.

KEW, M.C., BERGER, E.L. & KOPROWSKI, H. (1987). Carbohydrate antigen 19-9 as a serum marker of hepatocellular carcinoma: comparison with alpha-foetoprotein. *Br. J. Cancer*, **56**, 86.

KNEEBONE, R.L. & MANNELL, A. (1985). Cancer of the oesophagus in Soweto. *S. Afr. Med. J.*, **67**, 839.

KOPROWSKI, H., HERLYN, M., STEPLEWSKI, Z. & SEARS, H.F. (1981). Specific antigen in serum of patients with colon carcinoma. *Science*, **2**, 53.

MACH, J.P., JAEGER, P., BERTHOLET, M.M., RUEGSEGGER, C.H., LOOSLI, R.M. & PETTAVEL, J. (1974). Detection of recurrence of large bowel carcinoma by radioimmunoassay of circulating CEA. *Lancet*, **ii**, 535.

MAYER, R.J., GARNICK, M.B., STEELE, G.D. & ZAMCHECK, N. (1978). CEA as a monitor of chemotherapy in disseminated colorectal cancer. *Cancer*, **42**, 1428.

McGLASHEN, N.D. (1988). Oesophageal cancer in the black peoples of South Africa 1980–1982. *S. Afr. J. Sci.*, **84**, 92.

McINTIRE, K.R., WALDMAN, T.A., MOERTEL, C.G. & GO, V.L.W. (1975). Serum alpha-foetoprotein in patients with neoplasms of gastrointestinal tract. *Cancer Res.*, **35**, 991.

MELISSAS, J., WINTERS, Z. & MANNELL, A. (1983). CEA in carcinoma of the oesophagus. *S. Afr. J. Surg.*, **21**, 168.

MUÑOZ, N., CRESPI, M., GRASSI, A., WANG GUO QING, SHEN QIONG & LI ZHANG CAI (1982). Precursor lesions of oesophageal cancer in high risk populations in Iran and China. *Lancet*, **i**, 877.

RITTS, R.E., DEL VILLANO, B.C., GO, V.L.W., HEBERMAN, R.B., KLUG, T.L. & ZURAWSKI, V.R. (1984). Initial clinical evaluation of an immunoradiometric assay for CA19-9 using the NCI serum bank. *Int. J. Cancer*, **33**, 339.

STEINBERG, W.M., GELFAND, R., ANDERSON, K.K. and 4 others (1986). Comparison of the sensitivity and specificity of the CA19-9 and CEA assays in detecting cancer of the pancreas. *Gastroenterology*, **90**, 343.

TOROSIAN, M.H. (1988). The clinical usefulness and limitations of tumour markers. *Surg. Gynecol. Obstet.*, **166**, 567.

VUGRIN, D., FRIEDMAN, A. & WHITMORE, W.F. (1984). Correlation of serum tumour markers in advanced germ cell tumours with responses to chemotherapy and surgery. *Cancer*, **53**, 1440.

WAHREN, B., HARMENBERG, J., EDSMYR, F., JAKOBSSON, P. & INGIMARSSON, S. (1979). Possible tumour markers in patients with oesophagus cancer. *Scand. J. Gastroenterol.*, **14**, 361.

YANG, C.S. (1980). Research on oesophageal cancer in China: a review. *Cancer Res.*, **40**, 2633.